# Dose-Sparing Topical Administration: FK506-Loaded Nano-Micelles Achieve Efficient Therapy in a Murine Model of Vernal Keratoconjunctivitis

**DOI:** 10.3390/ph19060826

**Published:** 2026-05-25

**Authors:** Zhen Liang, Ping Lu, Yuan Tao, Zhen Zhang, Fei Song, Huiyun Xia, Jijun He, Xiaping Yao, Fudan Dong, Junjie Zhang, Guojuan Pu, Tianyang Zhou

**Affiliations:** Henan Eye Hospital, Henan Provincial People’s Hospital, People’s Hospital of Zhengzhou University, No. 7 Weiwu Road, Zhengzhou 450003, China; liangzhen@zzu.edu.cn (Z.L.); luping806176874@126.com (P.L.); ydunnn0429@163.com (Y.T.); cpu_zz@163.com (Z.Z.); songfei_0815@zzu.edu.cn (F.S.); xiahuiyun@163.com (H.X.); hjj1221@163.com (J.H.); yaoxiaping@163.com (X.Y.); 13125557162@163.com (F.D.); zhangjunjie@zzu.edu.cn (J.Z.)

**Keywords:** vernal keratoconjunctivitis, tacrolimus, nanomicelles, dose-sparing, pharmacokinetics, pharmacodynamics

## Abstract

**Background/Objectives**: Vernal keratoconjunctivitis (VKC) is a chronic, recurrent allergic disease with the risk of permanent injury or visual disabilities. Tacrolimus (FK506) is a potent immunosuppressant with insoluble ability and a high molecular weight. **Methods**: To address this disease, we successfully prepared FK506-loaded polymeric micelles (0.01%, FK506-MS) by a simple, organic solvent-free method. The physicochemical properties of FK506-MS were characterized. Corneal permeability, biocompatibility, and bioavailability were evaluated in vitro and in vivo in comparison with a commercially available FK506 suspension (0.1%, FK506-Susp). Therapeutic efficacy was also assessed in a murine model of VKC. **Results**: FK506-MS exhibited a small, homogeneous particle size with near-neutral surface charge. FK506-MS displayed a rapid and sustained release, along with excellent biocompatibility and stability. Ocular pharmacokinetic studies in rabbits revealed that FK506-MS, despite being only one-tenth the concentration of FK506-Susp, could achieve sufficient concentration in the conjunctiva with a prolonged half-life (T1/2) while systemic exposure in blood was markedly reduced. FK506-MS elicited comparable therapeutic responses across evaluated parameters: clinical symptoms, molecular biomarkers of inflammation, and histopathological findings. **Conclusions**: The dose-sparing advantage of FK506-MS suggests that the conventional paradigm of concentration-dependent therapeutic efficacy may require further refinement. The nanomicellar delivery system not only overcomes the solubility limitation of FK506 but also exhibits a potential therapeutic paradigm: achieving comparable clinical efficacy with a lower dose and reduced systemic exposure. These results provide a promising preclinical basis for the potential development of a topical tacrolimus therapy that may offer improved safety, cost-effectiveness, and patient adherence.

## 1. Introduction

Vernal keratoconjunctivitis (VKC) is a chronic, severe allergic conjunctivitis characterized by bilateral ocular inflammatory disorder and predominantly affecting children and young adults [1]. It reported that 4.0–11.1% of children and adolescents suffer from VKC in African countries, and a prevalence of 1.2% has been described in Japan [2]. Conventional management strategies, including antihistamines, corticosteroids, and mast cell stabilizers, are generally effective with significant side effects, especially with long-term use [3]. For instance, topical long-term use of corticosteroids leads to infectious episodes and elevated intraocular pressure (IOP). Even in children and adolescent patients, it has been reported that the incidence of steroid-induced glaucoma is significantly higher than that in adults, nearly to 2% [4,5]. Consequently, the pursuit of safer, yet equally effective therapeutic strategies is of critical importance.

Tacrolimus (FK506) acts by inhibiting calcineurin (CN) via binding to FK506 binding protein (FKBP) [6]. This inhibition of CN suppresses the dephosphorylation of nuclear factor of activated T-cells (NFAT), leading to suppression of the interleukin-2 (IL-2), IL-4, IL-5, and interferon-γ, eventually inhibiting the proliferation of T-cells [7]. FK506 also inhibits the release of histamine and cytokine production from mast cells [8]. These mechanisms contribute to the effectiveness of FK506 in VKC because the pathogenesis of VKC involves T-lymphocyte activation and cytokine release [9]. Recent accumulating clinical evidence positioned topical FK506 as an effective steroid-sparing therapy for severe ocular surface inflammation, which is important for chronic eye diseases, such as VKC, that require long-term medication [10,11]. Furthermore, FK506 achieved faster symptom reduction and better objective sign improvement with VKC than cyclosporine and dexamethasone, even after cessation of treatment [12,13]. Despite this clinical evidence, the ophthalmic translation of FK506 is limited by its high molecular weight and poor water-solubility [14,15]. While the former hinders corneal permeability, the latter traditionally requires organic solvents that cause ocular irritation and reduce patient compliance [16,17]. Overcoming these barriers to achieve effective delivery of FK506 to ocular tissues via topical administration without inducing serious adverse reactions remains considerably challenging.

Various technologies, such as in situ gelling, cationic nanoemulsion, nanocapsules, nano-micelles, and contact lenses, have been developed to improve the ocular bioavailability of poorly water-soluble drugs while minimizing adverse effects [18,19,20,21,22]. Among these, nano-micelles, specifically, polymeric micelles, represent a promising ocular drug delivery platform due to their advantages, such as high drug loading, sustained drug release, increased solubility, permeability across ocular barriers, stability, and bioavailability with minimal ocular toxicity [23,24,25]. Polymeric micelles exhibit a spherical shape and typically range in size from 10 to 100 nm [26], allowing for enhanced penetration into corneal and conjunctival cells, enabling the delivery of high concentrations of therapeutic molecules to ocular tissues, and improving bioavailability [27]. This approach holds promise as a dose-sparing strategy for FK506, given that potent immunosuppressive activity is observed in vitro at concentrations as low as 0.5 ng/mL [25], 0.005% FK506 eye drops have proven anti-inflammatory in patients suffering from rhinoconjunctivitis [14], and no direct proportionality between the increase in therapeutic effect and the dose [28]. On the other hand, topical 0.03% and 0.1% FK506 ointment induced corneal toxicity with epithelial defects in mice [26], and high-dose topical FK506 suspension frequently causes burning and irritation [29]. Thus, considering receptor saturation, the higher concentration of FK506 in the formulation might not be the best choice. So a low-concentration nano-micellar formulation of FK506 was designed to improve solubility and optimize its therapeutic index by maximizing target tissues delivery while reducing dose- and concentration-dependent side effects. This represents a significant advantage for long-term management of severe VKC.

The present study aims to leverage the advantages of the micellar system and develop a low-concentration FK506-loaded nanomicellar solution eye drops (FK506-MS) using Solutol HS15, a non-ionic surfactant with a low molecular weight, which is recommended to be added to injection formulations [30]. Its relatively bulky lipophilic portion and intrinsic micellar-forming property might allow HS15 for better drug solubilization. Accordingly, the solubility of FK506 in HS15, the preparation, characterization, and stability of FK506-MS, in vitro drug release, biocompatibility, and ex vivo permeability were investigated. Then, the pharmacological effect and pharmacokinetics profile of FK506-MS were also evaluated.

## 2. Results

### 2.1. Effect of HS15 Concentration on the Solubility of FK506

**Figure 1:**
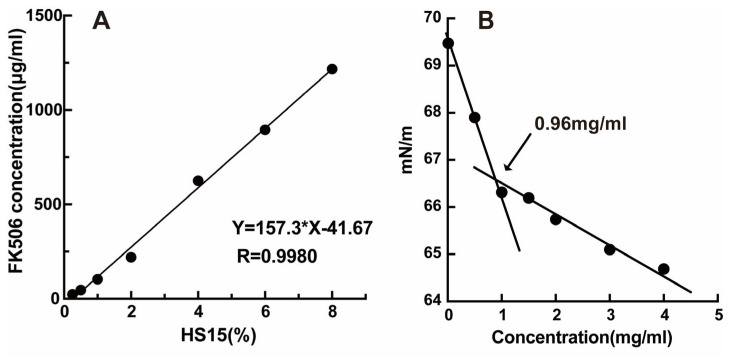
Solubility of FK506 in different concentrations of HS15 solution (**A**); surface tension versus concentration of HS15 (**B**).

The solubility of tacrolimus (FK506) in the medium free of HS15 was below the quantification limit of the HPLC-UV standard curve (5.875 μg/mL), confirming its poor aqueous solubility—a key barrier to ophthalmic formulation development. In contrast, the solubility of FK506 was significantly enhanced in aqueous solution of HS15, in a concentration-dependent manner, with a strong linear correlation between the solubility of FK506 and HS15 concentration (Figure 1). HS15 was chosen as the micelle-forming material instead of traditional organic solvents due to its FDA-approved ocular safety, low CMC for maintaining micellar integrity after tear dilution, and its ability to enhance corneal and conjunctival penetration. Specifically, when the dosage of HS15 is 0.9%, the solubility of FK506 can reach 0.01%. Thus, the dosage of HS15 in this prescription is 1%, above the CMC (about 0.096%) of HS15, which not only ensures adequate solubilization but also satisfies the design requirements for a stable ophthalmic nanodrug delivery system.

### 2.2. Preparation and Characterization of FK506-MS

#### 2.2.1. Physicochemical Properties of FK506-MS

**Figure 2:**
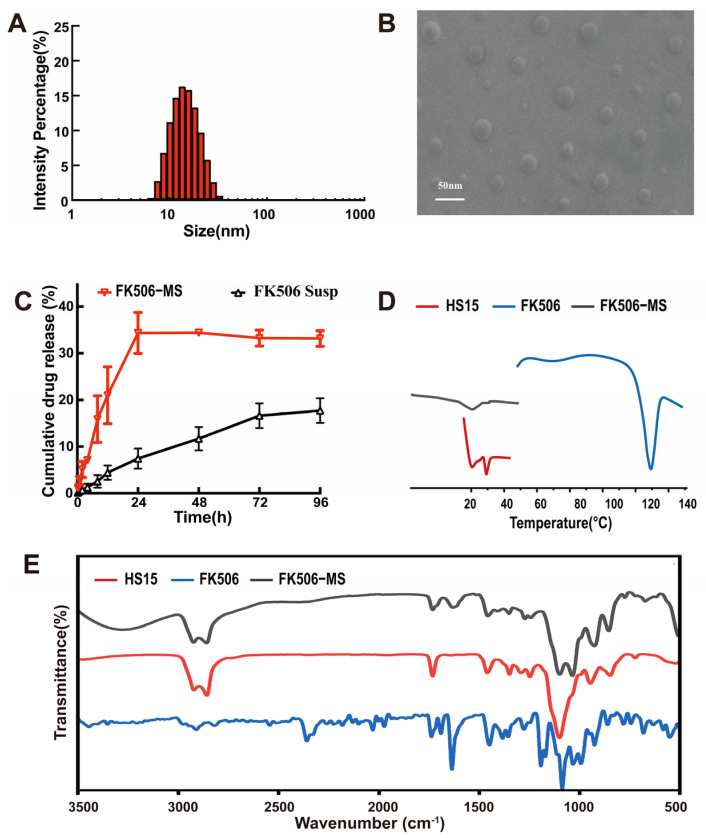
Characterization of FK506-MS. Size distribution of FK506-MS (**A**); SEM image of FK506-MS (**B**); In vitro release profiles of FK506-MS and FK506-Susp (**C**); DSC thermograms of FK506, HS 15 and FK506-MS (**D**); FT-IR spectra of FK506, HS 15 and FK506-MS (**E**).

FK506-loaded nanomicellar solution eye drops (FK506-MS) were successfully prepared by the direct dissolution method. As summarized in Table 1, the formulation had a pH of 5.40 ± 0.01 with high entrapment efficiency (EE). Dynamic light scattering (DLS) analysis showed the intensity size of FK506-MS was 13.63 ± 0.09 nm with a narrow size distribution (Figure 2A), which is consistent with the SEM observation and PDI results. The Polydispersity Index (PDI) was about 0.1, and scanning electron microscopy (SEM) images further confirmed the uniform spherical morphology of the micelles with good dispersibility (Figure 2B). The near-zero potential (0.15 ± 0.12 mV) of FK506-MS is an intrinsic characteristic of HS15 micelles. Its stability is attributed to the steric hindrance of the polyethylene oxide (PEO) shell, rather than electrostatic repulsion. The following long-term stability studies, including assessments of size and PDI, further corroborate this finding. This characteristic is instrumental in preventing irritation and inflammation associated with positively charged nanoparticles.

#### 2.2.2. In Vitro Drug Release

The drug release of FK506-MS and FK506 suspension (FK506-Susp) was shown in Figure 2C. FK506-MS depicted a significant advantage compared with FK506-Susp in the same release medium. FK506-MS showed a slow-release profile and entered a plateau period after 24 h. The release profile of FK506-MS was consistent with previous studies, showing an overall slow release related to the hydrophobic property of FK506 [31]. As shown in Table 2, the fitting results of the drug release kinetic model showed that FK506 release from the FK506-MS follows the first-order kinetic model with the highest correlation (*R^2^* = 0.98262). In the Korsmeyer–Peppas model, *n* of FK506-MS is 0.39 < 0.45, indicating drug release from FK506-MS via a Fickian diffusion mechanism (concentration-gradient-driven). In contrast, FK506-Susp exhibits non-Fickian transport [32].

#### 2.2.3. FT-IR Studies

Fourier transform–infrared spectra (FT-IR) were used to assess the formation of FK506-MS by analyzing the changes in typical peak positions, shape, and frequency. The pure HS15 displayed absorption peaks at 2859 cm^−1^ for C-H stretching, 1734 cm^−1^ for C=O stretching, and 1098 cm^−1^ for C-O-C stretching. while the pure FK506 displayed absorption peaks at 1244 cm^−1^ for O-CH3 stretching, 1637 cm^−1^ for C=C stretching, 1724 cm^−1^ for C=O stretching, 2925 cm^−1^ for C-H stretching, 2850–3000 cm^−1^ for -CH3/-CH2- stretching. However, the 1637 cm^−1^, 2925 cm^−1^, 2850–3000 cm^−1^ disappeared in the spectrum of the FK506-MS. This phenomenon indicates that FK506 is molecularly dispersed in an amorphous state within the hydrophobic core of the micelles [33] while FK506 exhibits a highly ordered crystalline lattice, which is consistent with previous research [34].

#### 2.2.4. Differential Scanning Calorimetry (DSC)

The DSC thermograms of the samples are presented in Figure 2D. The DSC thermograms of FK506 and FK506-MS showed sharp melting endotherms at 129.67 °C and 23.17 °C, respectively. HS15 showed sharp melting endotherms at 25.08 °C and 33.75 °C. However, the disappearance of the FK506 melting endotherm was observed in the thermograms of FK506-MS, consistent with FT-IR analysis, supporting the existence of FK506 in FK506-MS in an amorphous state [35].

#### 2.2.5. Ex Vivo Cornea Penetration Study

**Figure 3:**
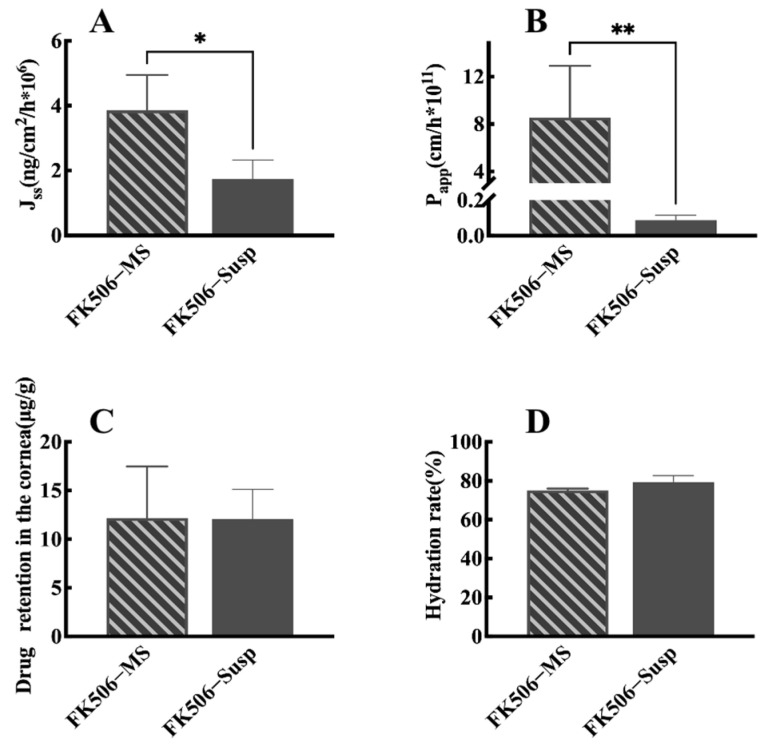
Drug corneal penetration profiles of FK506 formulations (n = 6, mean ± SD). Trans-corneal flux (J_ss_) (**A**); trans-corneal permeabilities (P_app_) (**B**); drug retention in cornea (**C**); and hydration rate of cornea (**D**). * Significantly (*p* < 0.05) different from the FK506-Susp, ** significantly (*p* < 0.01) different from the FK506-Susp.

The ex vivo corneal penetration studies of FK506-MS and FK506-Susp are shown in Figure 3. The trans-corneal flux (J_ss_) of FK506 from the FK506-MS was significantly higher than that of FK506 from the FK506-Susp (Figure 3A). The trans-corneal permeabilities (P_app_) of FK506 from the FK506-MS were about 100-fold that of FK506-Susp (Figure 3B). There was no difference in hydration rate and retention of FK506 in the cornea between FK506-MS and FK506-Susp.

### 2.3. Stability Studies

#### 2.3.1. Long-Term Stability Storage

**Figure 4:**
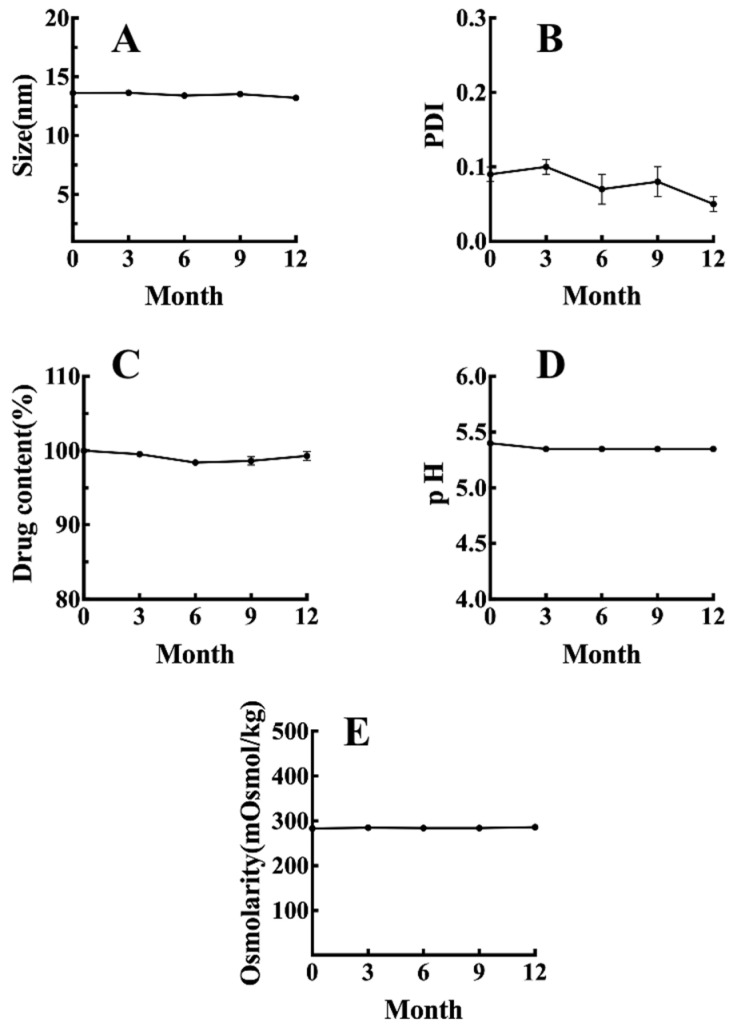
Size (**A**); PDI (**B**); drug content (**C**); pH (**D**); and osmolarity (**E**) of FK506-MS during 12 months at 4 °C.

Stability studies represent an important issue for the applicability of formulation in daily practice and industrial production. The stability of FK506-MS was monitored during 12 months at 4 °C (Figure 4). The key quality attributes of FK506 barely changed, indicating FK506-MS were stable once stored at 4 °C.

#### 2.3.2. In-Use Stability

As Table 3 displayed, drug content, osmolarity, and pH of FK506-MS were stable with respect to in-use stability conditions, and no contamination was detected in any of the evaluated conditions, indicating FK506-MS was stable during daily practice.

### 2.4. Biocompatibility Evaluation of FK506-MS

#### 2.4.1. In Vitro Cell Viability Test

**Figure 5:**
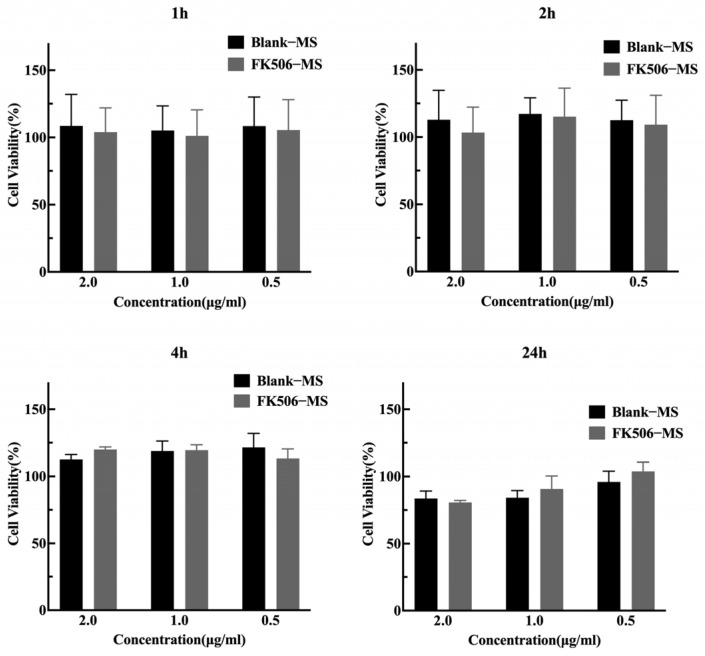
The percentage cell viability of FK506-MS and Blank-MS.

The cell toxicity of FK506-MS was evaluated by the CCK-8 assay. Cell viability was determined after FK506-MS and blank FK506-MS were incubated with human corneal epithelial cells (HCE-2, ACTT) at different time points. As Figure 5 displays, FK506-MS is safe for HCE-2 owing to a high cell viability percentage (>80%).

#### 2.4.2. Ex Vivo Hen’s Egg Test Chorioallantoic Membrane (HET-CAM)

**Figure 6:**
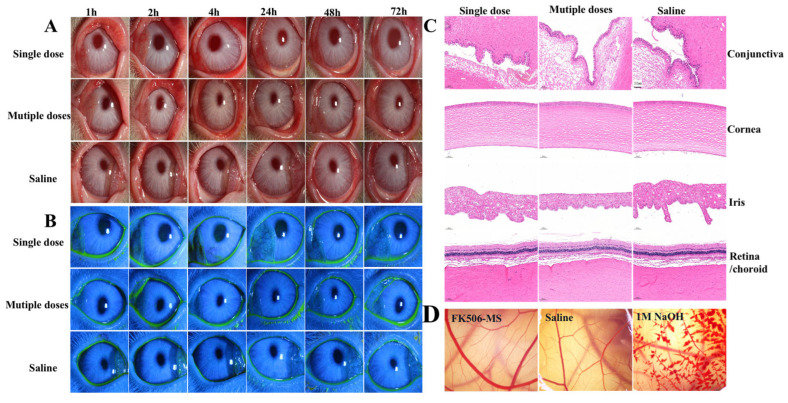
Ocular irritation test of FK506-MS. Ocular topical reaction of rabbits under visible light (**A**) and cobalt blue light (**B**); histopathological analysis of conjunctiva, cornea, iris, and retina/choroid (**C**); effect of controls and FK506-MS on the surface of the chorioallantoic membrane (CAM) (**D**).

The HET-CAM test revealed that FK506-MS behaved as the negative control (Saline) without inducing bleeding, lysis, or coagulation at the time of the study, as displayed in Figure 6D, which means that FK506-MS can be considered non-irritant.

#### 2.4.3. In Vivo Ocular Irritation Test

The ocular irritation of FK506-MS for a single dose and multiple doses was evaluated in New Zealand white rabbits. Compared with the Saline group, no abnormalities were observed in rabbit eyes (Figure 6A,B) and in the conjunctiva, cornea, iris, retina/choroid (Figure 6C).

### 2.5. In Vivo Pharmacokinetic Studies in Rabbits

**Figure 7:**
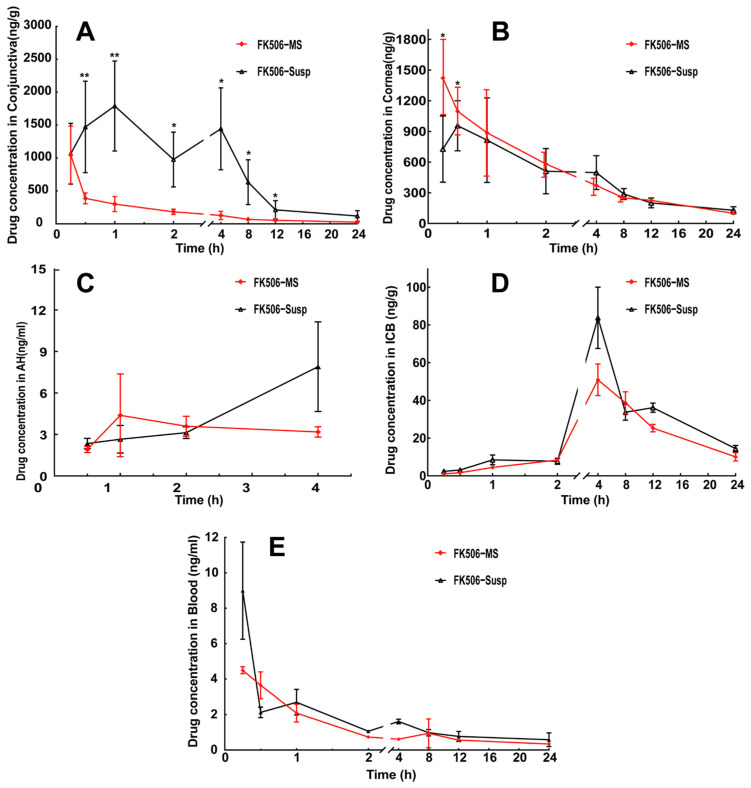
Drug concentration of different FK506 formulations in conjunctiva (**A**), cornea (**B**), AH (**C**), ICB (**D**), and blood (**E**) after single administration (mean ± SD, n = 6). FK506-MS: 0.01% FK506 micellar solution eye drops; FK506-Susp: 0.1% marketed FK506-Suspension (Talymus^®^); ** *p* < 0.01, * *p* < 0.05 vs. FK506-Susp.

The FK506 concentrations in the conjunctiva, cornea, AH, ICB, and blood versus time after one single dose of FK506-MS and FK506-Susp topical administration are shown in Figure 7. The FK506 concentrations of FK506-Susp in the conjunctiva were significantly higher than those of FK506-MS with a large variance. While this trend disappears in the cornea, the FK506 concentrations of FK506-MS in the cornea were significantly higher than those of FK506-Susp at 15 and 30 min. The FK506 could be detected at 30 min and could not be detected after 4 h in AH. The C_max_ of FK506-Susp in ICB was 1.6 times that of FK506-MS. As Table 4 displayed, the T_1/2_ of FK506-MS was 1.57 and 1.42 times that of FK506-Susp in the conjunctiva and cornea, respectively. However, the AUC_0–24h_ of FK506-MS was less than that of FK506-Susp in conjunctiva, cornea, AH, and ICB. However, both FK506 formulations were immediately absorbed into the blood to reach maximum concentration (C_max_) at 15 min, and the FK506 concentration remarkably decreased in the following hours. However, as Table 4 displayed, the peak concentration of FK506-MS was only half that of FK506-Susp, and its bioavailability was lower than that of FK506-Susp in the blood.

### 2.6. Pharmacodynamics

#### 2.6.1. Clinical Evaluation

**Figure 8:**
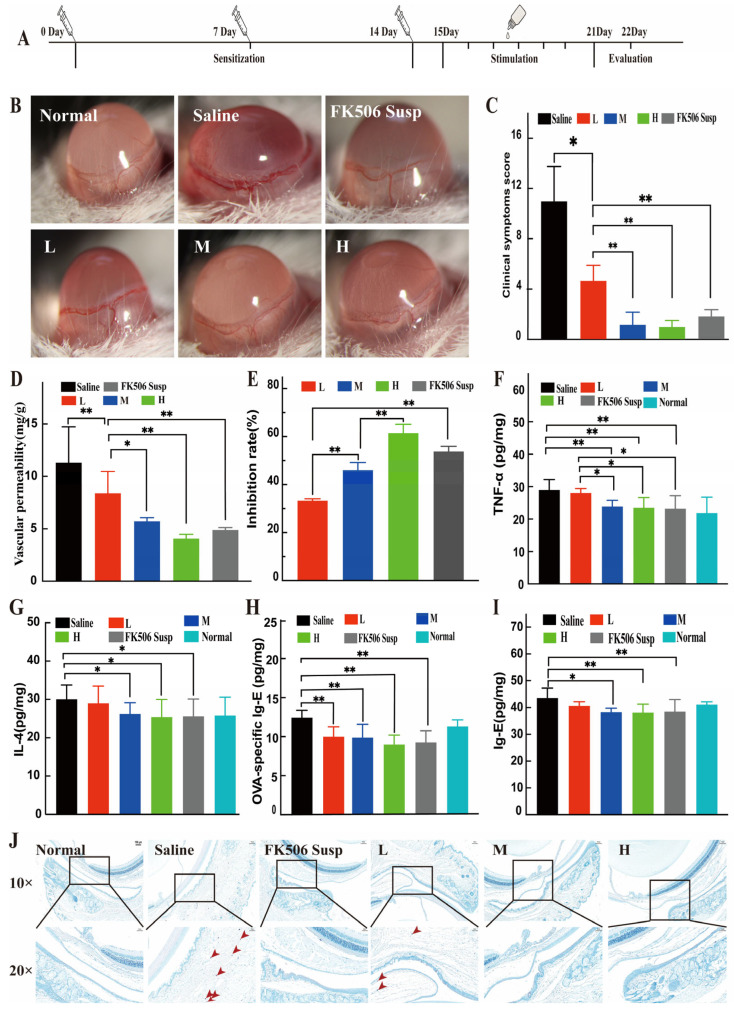
A schematic diagram summarizing the experimental workflow of pharmacodynamics (**A**). Representative images (**B**), clinical symptoms score (**C**), Evans Blue extravasation (**D**), inhibition rate (**E**), TNF-α (**F**), IL-4 (**G**), OVA-specific Ig-E (**H**), Ig-E (**I**), and mast cell (**J**) of mice in different groups after being challenged by OVA. Mast cells are indicated by red arrows. L, M, and H represent low, medium, and high concentrations of FK506-MS, respectively. ** *p* < 0.01, * *p* < 0.05.

The therapeutic effect of FK506-MS was assessed in the AC model. As Figure 8B displayed, severe conjunctival edema and congestion were found in mice of the saline group, and most of the eyelids were mostly closed with increased secretion around the eyes in the saline group, while slightly mild conjunctival edema and congestion were detected in the mice of the L group, while no secretion appeared around the eyes. The clinical symptoms score of the L group is significantly less than that of the saline group, while that of M, H, and FK506-Susp groups was significantly less than that of the L group (Figure 8C).

#### 2.6.2. Ocular Vascular Permeability Evaluation

The clinical symptoms scores and Evans Blue extravasation of the L group are significantly less than those of the saline group, while those of M, H, and FK506-Susp groups were significantly less than those of the L group (Figure 8C,D). However, the inhibition rate of M, H, and FK506-Susp groups was significantly higher than that of the L group, while the inhibition rate of the H group was significantly higher than that of the M group (Figure 8E).

#### 2.6.3. Enzyme-Linked Immunosorbent Assay (ELISA) Analysis

ELISA results indicated that levels of TNF-α (Figure 8F), IL-4 (Figure 8G), OVA-specific Ig-E (Figure 8H), and total Ig-E (Figure 8I) were significantly lower in the M, H, and FK506-Susp groups than in the Saline group. Among these four factors, only TNF-α was significantly lower in the M, H, and FK506-Susp groups compared with the L group. Additionally, the L group exhibited a significant reduction in OVA-specific Ig E compared to the Saline group.

#### 2.6.4. Histological Analysis

As shown in Figure 8J, many mast cells were observed in the saline group (indicated by red arrows), several mast cells were observed in the L group, while no mast cells were observed in the M, H, and FK506-Susp group.

## 3. Discussion

We successfully developed a nano-micellar solution-type eye drops of FK506 (FK506-MS) by the direct dissolution method, with the advantages of simplicity and organic solvent-free. Unlike conventional suspensions that require shaking before use and may cause transient blurred vision, FK506-MS was a homogenous and transparent solution with a high EE, small size, long-time stability, good biocompatibility, and sustained drug release. FK506 release from the FK506-MS best fits the first-order kinetic model, indicating that the drug is released after dissociation of the micelle structure and then diffuses into the dissolution medium from the dialysis bag [36,37]. These results were consistent with Gambogic acid release from lecithin/HS15 mixed micelles [38]. However, establishing in vitro-in vivo correlations (IVIVC) to predict in vivo performance still has limitations, because the conventional large-volume sink release model, such as 100 mL, poorly mimics the physiological conditions of the tear film [39]. Future work should incorporate biorelevant release models, such as small volume flow-through cells or microfluidic devices with simulated tear flow, to enable more meaningful IVIVC for ocular topical formulations.

In addition, the sustained drug release is imperative to maintain a minimally toxic, but therapeutic level of drug at the disease site for a prolonged period [40]. No ocular irritation (Figure 6) was observed, and FK506-MS exhibited high cell viability (Figure 5) at the highest concentration (2 μg/mL), exhibiting excellent biocompatibility [41]. Furthermore, the corneal hydration rate was normal in the ex vivo study [42]. Most notably, the J_ss_ and P_app_ of FK506-MS were significantly higher than those of FK506-Susp, which may be due to the small size of FK506-MS facilitating barrier crossing and active permeation enhancement of HS15 [43,44]. However, the drug was only detected at certain time points in AH in the pharmacokinetic study, perhaps due to the drug being quickly cleared away by the tear film [45], which is a noteworthy difference between ex vivo and in vivo studies.

Despite the concentration of active pharmaceutical ingredient of FK506-MS being only 1/10 of FK506-Susp, its safety and high efficiency in the treatment of VKC were verified through systemic pharmacokinetic and pharmacodynamic studies.

As Figure 7A displayed, FK506 concentrations and AUC in FK506-Susp were significantly higher than those of FK506-MS, leading to an increase in the systemic absorption (Figure 7E), potentially due to blood and lymphatic vessels present in the conjunctival tissue [46]. In contrast, the micellar formulation, characterized by its nanoscale particle size and hydrophilic surface, is more susceptible to diffusion and clearance, ultimately resulting in a reduced overall exposure [47]. Various clinical and non-clinical studies claimed that FK506 exposure was related to the incidence of adverse effects, such as neurologic side effects, diabetes mellitus, kidney and liver toxicity, after organ transplantation [48,49,50,51]. Therefore, reduced systemic absorption indicates reduced side effects. However, pharmacokinetic analysis revealed that the elimination half-life (T_1/2_) of FK506-MS in the conjunctiva was longer than that of FK506-Susp. This ostensibly paradoxical phenomenon could be elucidated by the fact that micelles could enhance the lipophilic drugs’ transport in and across the conjunctival epithelium due to their higher mobility, an enhanced interaction with the conjunctival epithelium, and, possibly, the penetration of intact micelles [46].

Subsequently, a mouse model of VKC was established to evaluate the efficacy of FK506-MS on VKC according to our previous study [52]. The model closely resembles human physiology, so its ocular vascular permeability and clinical symptoms are comparable with the human counterpart.

In our study, though the drug level in conjunctival tissues is significantly lower in the FK506-MS group than the FK506-Susp group at corresponding time points in the conjunctiva, respectively, it was still above the clinical therapeutic level [53]. As demonstrated in our study, this inhibition results in a significant reduction in the secretion of IL-4 (Figure 8G), a critical cytokine driving the Th2-type immune response. The attenuation of Th2 signaling consequently suppressed the downstream production of OVA-specific Ig-E (Figure 8H) and total Ig-E (Figure 8I), thereby diminishing the sensitization signal required for mast cell activation. Histopathological examination further revealed a significant decrease in mast cell infiltration within the conjunctival tissue (Figure 8J). These findings were consistent with previous reports [6,8]. Moreover, FK506-mediated suppression of macrophage activation resulted in a pronounced decrease in the release of the pro-inflammatory cytokine TNF-α (Figure 8F), which alleviates the terminal inflammatory cascade and associated tissue injury. This suppressive effect is documented in models of corneal neovascularization [54]. This multi-level mechanism, which encompasses the initial suppression of T-cell activity and culminates in the regulation of effector cell responses, aligns well with the established pharmacological profile of FK506.

Despite these encouraging findings, the comparison was performed at unequal drug concentrations (0.01% FK506-MS vs. 0.1% FK506-Susp). This 10-fold difference was deliberately chosen to evaluate the dose-sparing concept, whether a micellar formulation can achieve comparable therapeutic efficacy at a substantially reduced drug concentration. Nevertheless, the unequal dosing inevitably introduces potential bias, as the observed pharmacokinetic and pharmacodynamic outcomes may reflect dose-dependent pharmacodynamics rather than formulation superiority alone. Future studies employing matched concentrations are required to definitively confirm the delivery advantages of the micellar platform. Accordingly, our conclusions focus on the dose-sparing potential of FK506-MS rather than claiming inherent superiority over the commercial product.

Beyond our micellar system, several alternative strategies have been developed, including polymer-free microspheres, in situ gelling eye drops, nanocapsules, cubosomes, and cationic liposomes, aimed at treating ocular inflammatory diseases [20,22,54,55,56]. Compared with these systems, FK506-MS demonstrates several advantageous properties, such as a small particle size, long-time stability, and good biocompatibility. Importantly, all raw materials used in the preparation of FK506-MS are FDA-approved, and the direct dissolution preparation method is simple, scalable, cost-effective, and amenable to industrial translation.

## 4. Materials and Methods

### 4.1. Materials and Animals

Tacrolimus (FK506) was obtained from Sinopharm Chuankang Pharmaceutical Co., Ltd. (Chengdu, China). Ascomycin, as an internal standard (IS), was obtained from Dalian Meilunbio Technology Co., Ltd. (Dalian, China). HS15 was purchased from BASF SE (Ludwigshafen, Germany). The commercially available FK506-Suspension (FK506-Susp, TALYMUS^®^, Senju Pharmaceutical Co., Ltd., Osaka, Japan) was purchased from the local pharmacy. Ovalbumin (OVA), Dithiothreitol (DTT), and Evans Blue (EB) were purchased from Sigma-Aldrich. FAS eyeball fixative was obtained from Wuhan Servicebio Technology Co., Ltd. (Wuhan, China). All other reagents were of analytical grade, except for those employed in UPLC-MS/MS and HPLC assay, which were of MS and HPLC grade, respectively. Enzyme-linked immunosorbent assay (ELISA) kits for cytokine determination were obtained from MEIMIAN Biotech Co., Ltd. (Yancheng, China).

New Zealand white rabbits, weighing 2.0~2.5 kg (3 months of age) and Balb/c mice, weighing 18~22 g (aged 6~8 weeks), free of eye diseases, were used, which were obtained from Huaxing Laboratory Animal Company (Zhengzhou, China). All animal care and experimental protocols were approved by the Ethical Committee of Experimental Animal Care of Henan Eye Institute (HENNCA-2020-03, HENNCA-2020-08, and HENNCA-2020-04) and complied with National Institutes of Health guidelines. All procedures in the study conformed to the Association for Research in Vision and Ophthalmology Statement for the Use of Animals in Ophthalmic and Vision Research.

### 4.2. Solubility of FK506 in HS15

Excess FK506 was added to HS15 at concentrations of 0.25, 0.5, 1.0, 2.0, 4.0, 6.0, and 8.0% (*w*/*v*). The mixture was vortexed for 5 min and then incubated in a constant-temperature shaker at 25 °C and 150 r/min for 72 h [57]. Following centrifugation at 14,000 rpm for 30 min, the supernatant was collected, diluted, and injected into the HPLC system for the detection of FK506 concentration.

### 4.3. Preparation and Characterization of FK506-MS

#### 4.3.1. Preparation of FK506-Loaded Nanomicellar Solution Eye Drops (FK506-MS)

FK506 (0.1 g) was completely dissolved in the prescribed amount of HS15 with stirring at 35 °C. Subsequently, water for injection, maintained at the same temperature and containing relevant excipients, was added dropwise to the solution. The resulting mixture was then made up to the required final volume at room temperature, followed by sterile filtration.

Blank micelles, without FK506, were prepared using the same method as FK506-MS, excluding FK506 addition.

#### 4.3.2. Charicterization of FK506-MS

Particle size, Polydispersity Index (PDI), and Zeta potential of FK506-MS were determined by dynamic light scattering (Zetasizer, NanoZS90, Worcester, UK) after suitable dilution. The viscosity of FK506-MS was measured by a viscometer (model DV-II+Pro, Brookfield Engineering Laboratories, Inc., Middleboro, MA, USA). Critical micelle concentration (CMC) of HS15 was determined using a drop-shape analyzer (KRussDSA25, DKSH, Shanghai, China). Each value in the results was the average of three measurements.

#### 4.3.3. Entrapment Efficiency (EE%)

The entrapment efficiency (EE%) of FK506-MS was quantified by HPLC. Briefly, 5 mL FK506-MS was added to a 10 mL volumetric flask and diluted to the mark with acetonitrile and water (7:3 *v*/*v*). The concentration of FK506 (free drug) was separated by centrifugation at 4000 rpm for 20 min. Then the free drug in the filtrate in the centrifuge tube was determined by HPLC. The column was a reversed-phase C18 column (4.6 mm× 250 mm, 5 μm). The mobile phase consisted of deionized water and organic phase (65:35 *v*/*v*) at a detection wavelength of 220 nm. The flow rate was 1.5 mL/min, and the column temperature was maintained at 60 °C. EE was calculated as [58]

EE (%) = (W_0_ − W_1_) × 100/W_0_ (1)

where W_0_ is the weight of FK506 added, and W_1_ is the weight of FK506 free in the FK506-MS.

#### 4.3.4. In Vitro Drug Release

The in vitro drug release of FK506-MS and FK506 commercial suspension (FK506-Susp) was assessed [59]. 1 mL of FK506-MS and FK506-Susp was pipetted into a dialysis bag (MW cutoff: 3500 Da), which was then sealed at both ends. Then the dialysis bag was placed in 100 mL of release medium (artificial tear solution with 0.5% HS15) at 37 ± 0.5 °C under shaking to maintain sink conditions. Sample aliquots (1 mL) were withdrawn at predetermined time intervals, and an equivalent amount of fresh artificial tear solution was added to the dissolution medium. The drug concentrations were determined using the UPLC-MS/MS assay method described in Section 4.6.3.

#### 4.3.5. Morphology Analysis by Scanning Electron Microscopy

Surface morphology of FK506-MS was performed by scanning electron microscopy (SEM, Prisma E, Thermo Fisher, Waltham, MA, USA) after samples were rapidly frozen in liquid nitrogen, followed by freeze-drying for 24 h, and gold-coated for conductivity. Subsequently, samples were observed under SEM at an accelerating voltage of 10 kV.

#### 4.3.6. Fourier Transform–Infrared Spectra (FT-IR)

Spectral scanning of FK506-MS aqueous formulation, FK506, and HS15 was investigated by an AVATAR 370 FT-IR instrument (Alpha II, Bruker, Ettlingen, Germany). All samples were measured at 25 °C in the range of 4000–400 cm^−1^ [60]. The FT-IR spectra of these three samples were recorded with a resolution of 4 cm^−1^, and each spectrum was performed for 10 scans.

#### 4.3.7. Differential Scanning Calorimetry (DSC)

Thermograms of the different samples were obtained using a DSC Q200 instrument (TA Instruments, New Castle, DE, USA). Appropriate amount (2–3 mg) of pure FK506, HS15, and freeze-dried FK506-MS were placed in hermetically sealed aluminum pans, and a blank sealed aluminum pan was used as the reference. The thermogram was recorded at a heating rate of 4 °C·min^−1^ with nitrogen as a purging gas (0.2 MPa) over a temperature range of 20–300 °C.

#### 4.3.8. Ex Vivo Permeation Studies

Drug permeation studies were carried out using side-by-side diffusion cells by modifying the Franz diffusion cells [61]. The freshly excised rabbit corneas were clamped between the donor and the receptor chamber. 2 mL of each formulation (FK506-MS or FK506-Susp) and 3 mL of artificial tear solution were added to the donor chamber, while 5 mL of artificial tear solution was added to the receptor chamber. The cell top was completely sealed with parafilm to prevent evaporation. The cells were maintained at 37 ± 0.5 °C with magnetic stirring. The samples were collected at 15, 30, 60, 90, 120, 160, 180, and 240 min, and an equal volume of preheated fresh medium was replaced immediately. The samples were analyzed using the UPLC-MS/MS assay method described in Section 4.6.3. The analysis method had been previously validated. Sink conditions were maintained in the receptor compartment during ex vivo permeation studies.

The permeation enhancement was assessed by permeation parameters, and the FK506 flux at 240 min (J_ss_, μg/cm^2^/h) and permeability coefficient (P_app_; cm/h) were calculated.

J_ss_ = (ΔQ/Δt)/(A × 60) (2)

P_app_ = J_ss_/C_0_ (3)

where ΔQ/Δt is the steady-state flux into the receiving solution (μg/min), A is the area of exposed cornea (0.694 cm^2^), and C_0_ is the initial drug concentration added to the donor chamber.

After the last sampling, the corneas were rinsed, and the corneal hydration rate was calculated in Equation 4:

Hydration rate (%) = (1 − W_a_/W_b_) × 100 (4)

where W_a_ is the wet cornea weight, and W_b_ is the corresponding dry cornea weight after a desiccation of 8 h at 80 °C.

### 4.4. Stability Studies

#### 4.4.1. Long-Term Stability

The stability of the FK506-MS at pilot scale was assessed for 12 months at 4 °C according to ICH guideline Q1A (R2): Stability Testing of New Drug Substances and Products. Drug content, size, PDI, pH, and osmolarity of FK506-MS were observed at 0, 3, 6, 9, and 12 months. The size and PDI were measured as described in Section 4.3.2. The drug content was determined by HPLC, as described in Section 4.3.3. pH was measured with a pH meter (pHS-3C, INASE, Shanghai, China), and osmolality was determined by the freezing point depression method using an osmometer (STY-1A, Tianda Tianfa, Tianjin, China).

#### 4.4.2. In-Use Stability

The in-use stability of FK506-MS at pilot scale was assessed for 28 days at 4 °C according to the EMA guideline: Note for Guidance on In-Use Stability Testing of Human Medicinal Products. We simulated daily patient use by withdrawing 1–2 drops two times a day from the original multidose container. Drug content, osmolarity, pH, and sterility on day 0, 7, 14, 21, and 28. The methods for determining drug content, osmolality, and pH were as described in Section 4.4.1. Sterility testing was performed using the membrane filtration method according to the Chinese Pharmacopeia (ChP).

### 4.5. Biocompatibility Evaluation of FK506-MS

#### 4.5.1. In Vitro Cytotoxicity Assay

The in vitro cytotoxicity of FK506-MS was evaluated using human corneal epithelial cells (HCE-2, ACTT). Cell viability was determined according to the protocol described previously, with minor modifications [60]. Briefly, cells were seeded in 96-well plates at a density of 5 × 10^3^ cells/well and cultured in Dulbecco’s Modified Eagle Medium (DMEM)/F12 medium supplemented with 10% fetal bovine serum (FBS) and 1% penicillin-streptomycin double antibody at 37 °C with 5% CO_2_ for 24 h to allow full adhesion.

After cell adhesion, the original medium was discarded, and FK506-MS solutions with serial concentrations (0.5, 1, 2 μg/mL, calculated based on FK506 content) were added to the corresponding wells. Blank micelles (blank MS, without FK506 loading) at the same concentration gradient were set as control groups, with pure cell culture medium as the negative control. After co-incubation for 1 h, 2 h, 4 h, and 24 h, respectively, 10 μL of CCK-8 reagent was added to each well, followed by continuous incubation for 2 h. The absorbance value at 450 nm was measured using a microplate reader (PerkinElmer 2104 Multilabel Reader, Shanghai, China). Cell viability was calculated following the aforementioned reference [60].

#### 4.5.2. Ex Vivo Hen’s Egg Test on Chorioallantoic Membrane (HET-CAM)

The ex vivo ocular irritation effects of the FK506-MS were assessed via the HET-CAM assay [62]. Freshly fertilized White Leghorn chicken eggs from Jinan Spafas Poultry Co., Ltd. (Jinan, China) were incubated in a humidified bioclimatic incubator at 37 ± 0.5 °C. Throughout incubation, the eggs were rotated manually every 2 h during incubation to prevent embryo adherence to the shell membrane. On the 9th day of incubation, eggs were candled to confirm the development stage by observing the embryo and CAM blood vessels through the illuminated shell. The eggs were wiped with 70% ethanol and kept upright with the blunt end facing upwards. A small window was made by carefully removing the shell and inner membrane to expose the CAM. Test sample solutions (300 μL) were instilled on the exposed CAM. The reactions on CAM, such as hemorrhage, blood vessel lysis, and coagulation, were observed at different times over 5 min after installing the test solution and documented. NaCl solution (0.9% *w*/*v*) was used as a negative control and NaOH (1M) as a positive control.

#### 4.5.3. In Vivo Ocular Irritation Test

The ocular irritation effects of the FK506-MS with single-dose and multiple-dose administration were assessed following a modified Draize test [61]. Ten male rabbits (2.0–2.5 kg) free of ocular diseases were selected and randomly divided into two groups. A volume of 100 μL of the FK506-MS was instilled into the conjunctival sac in the right eye, while the left eye was treated with physiological saline as a control for single-dose evaluation. A volume of 100 μL of the FK506-MS was instilled into the conjunctival sac in the right eye twice a day for 28 days, while the left eye was treated with physiological saline as a control for multiple-dose evaluation. The corneal lesions and opacity (score 0–4), conjunctival chemosis, redness, discharge (score 0–3), and iris alterations (score 0–2) were microscopically observed at 1, 2, 4, 24, 48, and 72 h after the last instillation. Ocular irritation scores for each rabbit were calculated by adding together the irritation scores for the cornea, conjunctiva, and iris. The irritation severities were obtained by dividing the total scores for all rabbits by the number of rabbits. A positive reaction was defined when the average numerical score equaled or exceeded specific numerical cutoffs, such as corneal opacity score ≥ 1, iris score ≥ 1, or conjunctival score ≥ 2.

### 4.6. In Vivo Pharmacokinetic Studies in Rabbits

Forty-eight male rabbits (2.0–2.5 kg) free of ocular diseases were selected and randomly divided into two groups, with three rabbits per group at each time point. The test group received bilateral 50 μL of pre-sterilized FK506-MS into the conjunctival sac per eye, while the control group was administered a single dose of 50 μL of FK506-Susp via the same route for both eyes.

#### 4.6.1. Blood Collection and Drug Extraction

Upon reaching the predetermined time points, the rabbits from each group were euthanized by intravenous injection of 40 mg/kg pentobarbital sodium [63], and blood samples were collected via cardiac puncture. An aliquot (100 μL) of each whole blood sample was mixed with 10 μL of the IS working solution and 90 μL of 10% (*v*/*v*) aqueous methanol solution. After ultrasonication of the mixture while floating an icepack on water in the bath for 10 min, 10 μL of a 0.05 mol/L aqueous hydrochloric acid and 1 mL of methyl tert-butyl ether were added, and the resulting mixture was centrifuged at 8000 rpm for 10 min (Mini Spin plus; Eppendorf, hamburg, Germany). Subsequently, the supernatant was transferred to a 5 mL glass tube and dried under a nitrogen atmosphere at 60 °C. The resulting residue was reconstituted in 100 μL methanol, then vortexed for 1 min and centrifuged at 3000 rpm for 8 min (LD5-2A; Beijing Medical Centrifuge Company, Beijing, China). The supernatant was analyzed by UPLC-MS/MS analysis.

#### 4.6.2. Eye Tissue Collection and Drug Extraction

After blood collection, the rabbits were euthanized by intravenous injection of another overdose of pentobarbital sodium. Subsequently, the ocular surface of each rabbit was rinsed thoroughly with normal saline to eliminate residual drug formulations. Aqueous humor (AH) was withdrawn with a 26-G needle attached to a disposable syringe. Then, the conjunctiva, cornea, and iris-ciliary body (ICB) samples were harvested with surgical scissors and knives. All samples were rinsed with saline, gently wiped, weighed, and kept at −80 °C until used for extraction.

The conjunctiva, cornea, and ICB samples were thawed at room temperature and cut into scraps. Then, 990 μL of methanol and 10 μL of IS working solution were added and centrifuged for 10 min at 8000 rpm after being soaked for 24 h, respectively, at 4 °C. The supernatant was transferred to a sample vial for UPLC-MS/MS analysis. The AH was conducted following the method as reported [59].

To assess the bioavailability of FK506, the pharmacokinetic parameters of FK506 in the conjunctiva, cornea, AH, ICB, and blood were estimated using DAS2.1.1 software. Four main pharmacokinetic parameters were presented as the detected value: the maximum concentration (C_max_), the time (T_max_) for C _max_ to occur, the elimination half-life time (T_1/2_), and the area under the concentration-time curve up to 24 h (AUC_0–24h_).

#### 4.6.3. UPLC-MS/MS Analysis of FK506

An Acquity UPLC system equipped with an ultra-high-pressure pump system, an autosampler, and a temperature-controlled vial tray (Waters, Milford, CT, USA) was used for chromatographic separation. The mobile phase was 0.1% (*v*/*v*) formic acid aqueous solution-methanol (32:68, *v*/*v*) with isocratic elution at 0.2 mL/min. A reversed-phase chromatographic column (Xterra MS, C18, 3.5 μm, 2.1 mm × 50 mm, Waters) was maintained at 60 °C, and the autosampler was set at 4 °C. Injection volume was 4 μL.

Mass spectrometric detection was carried out by a Xevo TQ tandem quadrupole mass spectrometer (Waters, Milford, CT, USA) coupled with a Z-Spray electrospray ionization (ESI) source (positive ion mode, ES+). The capillary voltage was maintained at 4.0 kV. Ultrapure liquid nitrogen was used to supply the desolvation gas with a flow rate of 1000 L/h, and the desolvation temperature was set to 500 °C. The cone voltage was maintained at 12.0 V. The collision energy was set at 12.0 V, and argon was used as the collision gas at a flow rate of 0.15 mL/min.

Analytes were detected and quantified in multiple reaction monitoring mode with mass transitions of 821.3/768.5 *m*/*z* for FK506 and 809.8/756.1 m/z for IS. The data were acquired using MassLynx 4.1 software (Waters). The method was validated for specificity, sensitivity, linearity, recovery, matrix effect, precision, accuracy, stability, and dilution integrity before animal treatment. FK506 concentration in blood and ocular tissue was quantified by the IS method based on the peak area ratio of FK506 to IS.

### 4.7. Pharmacodynamics

#### 4.7.1. Vernal Keratoconjunctivitis Model [52,64]

Balb/c mice (18–22 g, 6–8 weeks old) free of ocular diseases were intraperitoneally injected with 200 μL of 1 mg/mL ovalbumen (OVA) and 40 mg/mL aluminum hydroxide on days 0, 7, and 14 for sensitization. On day 15, the mice were completely randomized into 5 groups with 15 per group, including the normal saline group, positive control group (FK506-Susp, Talymus^®^), FK506-MS high-dose group (0.02% FK506-MS, H), FK506-MS medium-dose group (0.01% FK506-MS, M), and FK506-MS low-dose group (0.005% FK506-MS, L). From days 15–21, the mice were challenged by topical instillation of 5 μL of 80 mg/mL OVA. After each OVA challenge, the corresponding eye drops were topically administered twice daily.

On day 22, the therapeutic efficacy of the drugs was evaluated. The allocation of mice in each group was as follows: 6 mice for ocular symptom observation and scoring, 6 mice for vascular permeability assessment, and 3 mice for histological examination. After clinical scoring, the mice were subjected to cardiac blood collection, followed by eyeball harvesting for subsequent use.

#### 4.7.2. Clinical Grading

On day 22, each eye was evaluated in a masked fashion by experienced observers under a slit lamp microscope after the animals were stimulated with OVA. The severity of VKC was graded based on these parameters, including conjunctival chemosis, conjunctival congestion, and discharge. Each parameter was scored on the criteria from 0~3 and the total clinical score was calculated as the sum of the scores for the three individual parameters.

#### 4.7.3. Ocular Vascular Permeability Evaluation

On day 22, each eye was treated with 1M DTT to eliminate the conjunctival barrier. After 15 min, 0.2 mL of the EB (2 mg/mL) solution was injected via the tail vein, and the mice were challenged immediately with 5 μL of 10% OVA. Then the mice were euthanized by excessive anesthesia with pentobarbital sodium after being challenged for 30 min. Eyeballs were harvested and immersed in 0.3 mL of 0.5% Na2SO4: acetone (3:7, *v*/*v*) at 40 °C for 24 h with continuous shaking. After centrifuging at 8000 rpm for 10 min, the absorbance of the supernatant was recorded at 620 nm. The inhibition rate was calculated as follows [65]:

I = (P − D)’100%/P

I represents the exudation inhibition rate, D denotes the average EB exudation amount of the drug-treated eyes, and P represents the average EB exudation of the eye treated with saline.

#### 4.7.4. Histological Analysis

On day 22, three mice were euthanized by excessive intraperitoneal injection of sodium phenobarbital. The whole eyeballs were carefully enucleated with the adjacent conjunctiva retained, and then immediately fixed in FAS, followed by standard toluidine blue staining and histopathological observations.

#### 4.7.5. Enzyme-Linked Immunosorbent Assay (ELISA) Analysis

On day 22, the eyeballs and whole blood were harvested after being challenged by OVA. The blood serum was extracted by centrifugation at 6000 rpm for 10 min at 4 °C. The level of IL-4 and TNF-α in ocular tissues, as well as Ig-E and OVA-specific Ig-E in serum, were quantified by ELISA in accordance with the manufacturer’s protocol.

### 4.8. Statistical Analysis

All data were expressed as means ± standard deviations (SDs). Results were analyzed by SPSS software (SPSS version 27, Chicago, IL, USA). Ex vivo permeation studies and in vivo pharmacokinetic studies were assessed using independent sample *t*-tests (2-sided). In a murine model of VKC, evaluation of clinical scores, vascular permeability, and levels of cytokine was achieved using one-way analysis of variance (ANOVA) followed by Tukey’s post hoc test. The significance level was set at *p* < 0.05.

## 5. Conclusions

Despite containing only one-tenth the drug concentration of the conventional suspension (FK506-Susp), FK506-MS maintained effective ocular surface drug levels and immunosuppressive activity in a murine VKC model while significantly reducing systemic exposure. This dose-sparing and improved safety suggest that FK506-MS is a promising preclinical candidate for topical ocular delivery of FK506. Further research should focus on clinical translation, mechanistic elucidation, and exploration of its therapeutic potential in other ocular immune-mediated diseases, such as corneal graft rejection.

## Figures and Tables

**Table 1:** Physicochemical properties of FK506-MS.

Intensity Size (nm)	PDI	Zeta Potential (mv)	pH	EE (%)	Viscosity (cP)
13.63 ± 0.09	0.09 ± 0.01	0.15 ± 0.12	5.40 ± 0.01	98.71	2.28 ± 0.13

**Table 2:** The mathematical models are fitting for FK506-MS and FK506-Susp.

	FK506-MS		FK506-Susp	
	Equation	*R^2^*	Equation	*R^2^*
Zero-order	Q = 8.68 + 0.35t	0.61207	Q = 0.92 + 0.20t	0.96195
First-order	Q = 34.59 × (1 × e^−0.083t^)	0.98262	Q = 22.97 × (1 × e^−0.016t^)	0.99612
Higuchi	Q = 4.04 × t^1/2^ + 1.80	0.82144	Q = 2.03 × t^1/2^−1.99	0.98268
Korsmeyer–Peppas	Q = 6.75 × t^0.39^	0.85477	Q = 0.72 × t^0.72^	0.98897

**Table 3:** The key quality attributes of FK506-MS during the in-use stability.

Time (Days)	Drug Content	pH	Osmolarity	Sterility
0	101.50 ± 1.61	5.5	289	Meet sterility requirement
7	101.00 ± 0.36	5.5	285	Meet sterility requirement
14	99.93 ± 0.35	5.5	287	Meet sterility requirement
21	101.10 ± 0.70	5.5	285	Meet sterility requirement
28	101.17 ± 0.87	5.5	285	Meet sterility requirement

**Table 4:** Pharmacokinetic parameters of eye drops in ocular tissues (mean ± SD, n = 6).

Pharmacokinetic Parameters		Formulations	
		FK506-MS	FK506-Susp
Conjunctiva	T_1/2_ (h)	11.15	7.10
	C_max_ (ng/mL)	1047.45 ± 437.04	1790.13 ± 683.55
	T_max_ (h)	0.25	1
	AUC_0–24h_ (ng/mL·h)	2144.28	12,861.95
Cornea	T_1/2_ (h)	10.56	7.43
	C_max_ (ng/mL)	1422.33 ± 361.07	956.15 ± 244.99
	T_max_ (h)	0.25	0.5
	AUC_0–24h_ (ng/mL·h)	6826.94	7130.28
Aqueous humor	T_1/2_ (h)	3.44	4.98
	C_max_ (ng/mL)	4.39 ± 2.99	7.90 ± 3.24
	T_max_ (h)	1	4
	AUC_0–24h_ (ng/mL·h)	12.27	14.65
ICB	T_1/2_ (h)	8.45	7.98
	C_max_ (ng/mL)	50.90 ± 20.47	83.76 ± 39.62
	T_max_ (h)	4	4
	AUC_0–24h_ (ng/mL·h)	587.59	782.00
Blood	T_1/2_ (h)	20.62	28.03
	C_max_ (ng/mL)	4.5 ± 0.20	9.00 ± 2.75
	T_max_ (h)	0.25	0.25
	AUC_0–24h_ (ng/mL·h)	17.36	27.57

FK506-MS: 0.01% FK506 micellar solution eye drops; FK506-Susp: 0.1% marketed FK506 suspension (Talymus^®^); C_max_: the maximum concentration; T_max_: the time for C_max_ to occur; T_1/2_: the elimination half-life time; AUC_0–24_: the area under the concentration-time curve up to 24 h.

## Data Availability

The data presented in this study are available on request from the corresponding author. The data are not publicly available, as these were originally produced through research.

## Abbreviations

The following abbreviations are used in this manuscript:

VKC	Vernal keratoconjunctivitis
FK506	Tacrolimus
FK506-MS	FK506-loaded polymeric micelles
FK506-Susp	Commercially available FK506 suspension
CN	Calcineurin
FKBP	FK506 binding protein
NFAT	Nuclear factor of activated T-cells
IL-2	Interleukin-2
IL-4	Interleukin-4
IL-5	Interleukin-5
IS	Internal standard
OVA	Ovalbumin
DTT	Dithiothreitol
EB	Evans Blue
PDI	Polydispersity Index
HET-CAM	Hen’s Egg Test on Chorioallantoic Membrane
ICB	Iris–ciliary body
AH	Aqueous humor
ELISA	Enzyme-linked immunosorbent assay
TNF-α	Interferon-α
